# Supplementary figures and images for: Epidemiological and genomic investigation of chikungunya virus in Rio de Janeiro state, Brazil, between 2015 and 2018
Source: PLoS Negl Trop Dis. 2023 Sep 28;17(9):e0011536. doi: 10.1371/journal.pntd.0011536 (PMC10564160; doi:10.1371/journal.pntd.0011536)

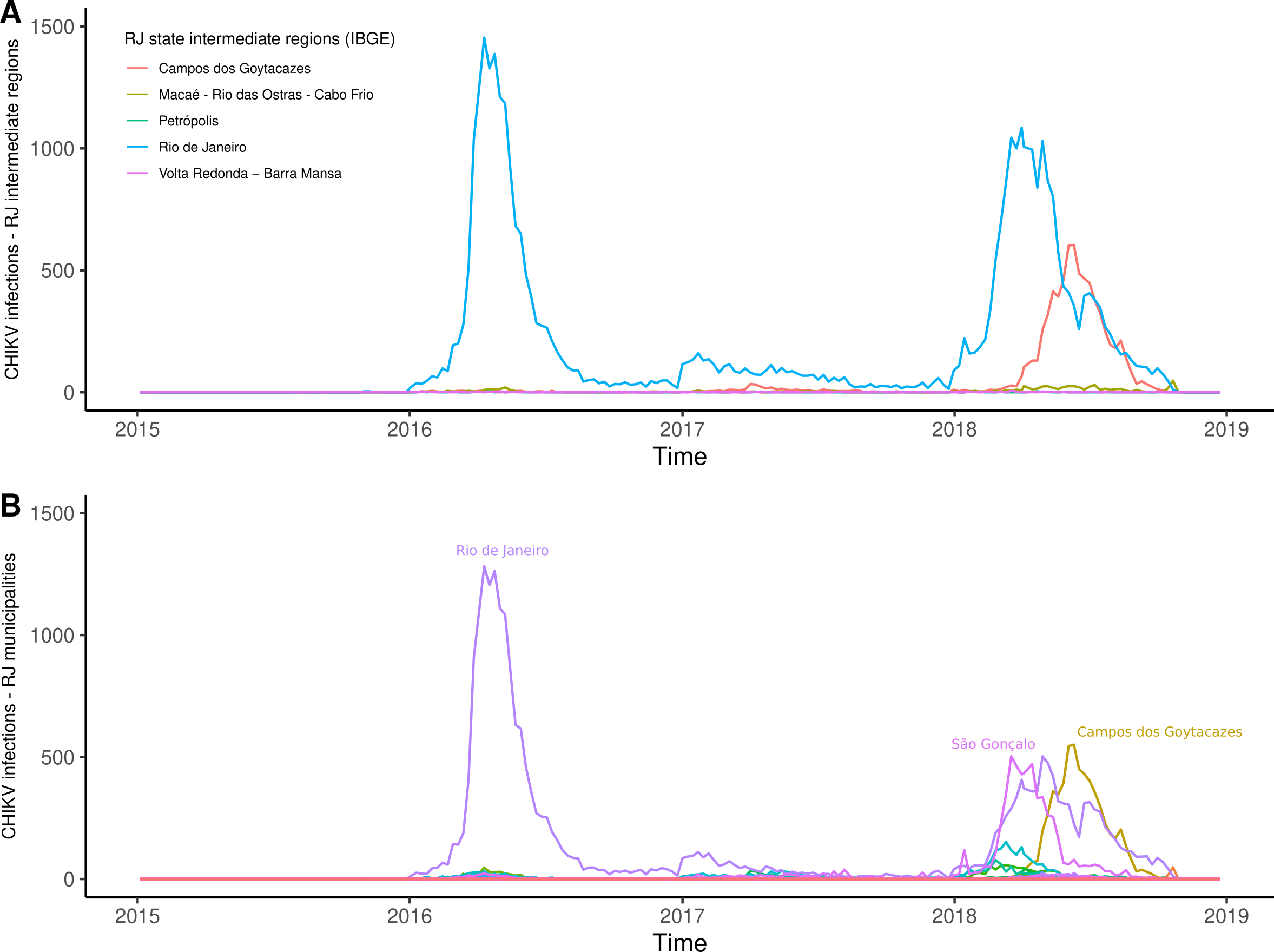

Supplement: S1 Fig — (A) CHIKV incidence per state region. Rio de Janeiro is officially divided into five intermediate regions, each comprehending between 12 and 26 municipalities (IBGE, https://www.ibge.gov.br/apps/regioes_geograficas/, last accessed 17 August 2022). (B) CHIKV incidence per municipality. As the state comprehends 92 municipalities, and many of them present low incidence levels through the entire period, individual color legends have been omitted. Municipalities that at any point presented weekly incidence above 500 cases were highlighted in the plot (Campos dos Goytacazes, Rio de Janeiro, São Gonçalo). (TIFF) [file pntd.0011536.s001.tiff]

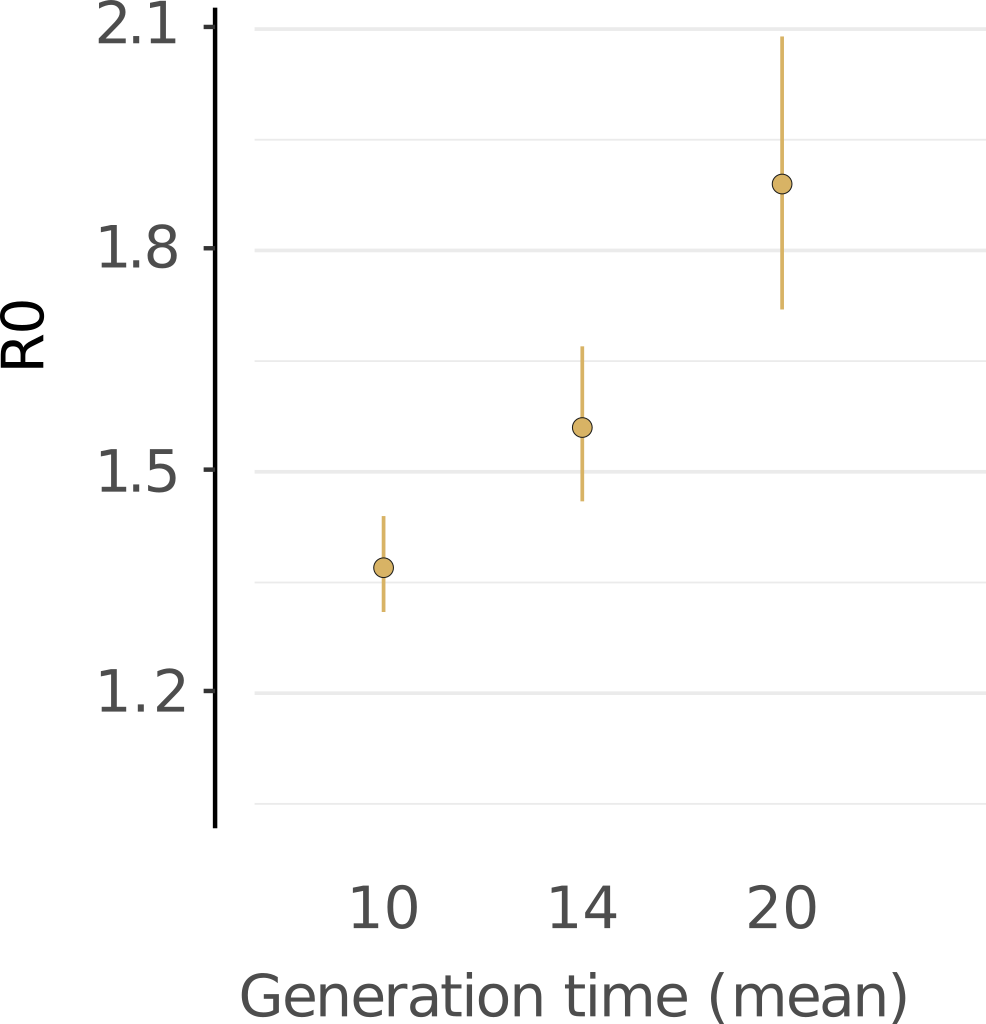

Supplement: S2 Fig — R0 estimates obtained with epidemiological data under different generation time distributions (gamma distributions with means 10, 14 and 20, and constant standard deviation: 6.4 days). (TIFF) [file pntd.0011536.s002.tiff]

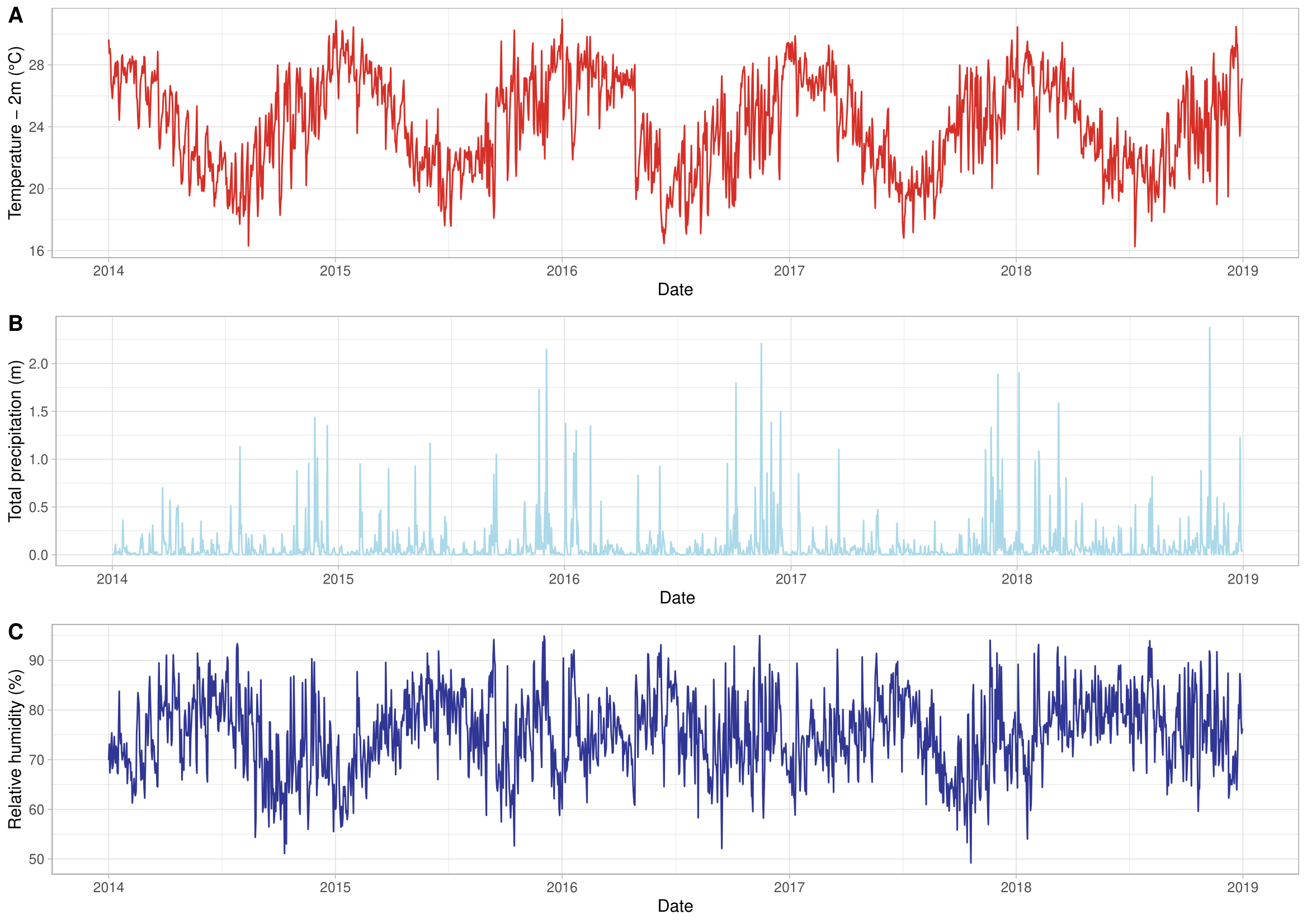

Supplement: S3 Fig — Time series of climatic variables—(A) 2m temperature, (B) total precipitation and (C) relative humidity—for Rio de Janeiro state between 2014 and 2018. All climatic data was obtained from the ECMWF reanalysis product [34]. (TIFF) [file pntd.0011536.s003.tiff]

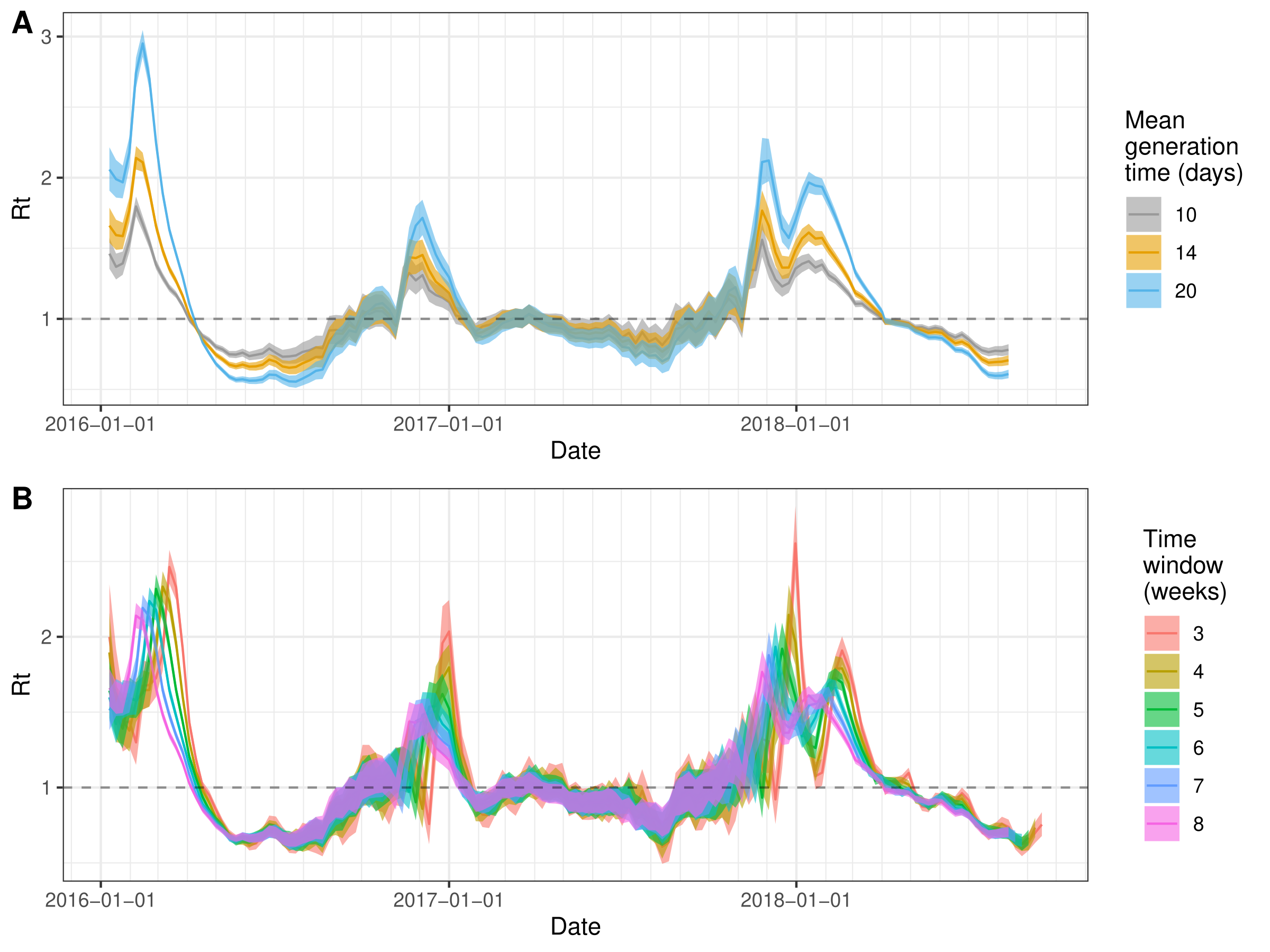

Supplement: S4 Fig — (A) Rt estimate performed with different generation time (GT) distributions (gamma distributions with means 10, 14 and 20, and constant standard deviation: 6.4 days). Colors indicate estimates for different GTs (10: gray, 14: yellow, 20: blue). (B) Rt estimates performed with different sliding window lengths (3 to 8 weeks). Colors indicate estimates for different window lengths (3: salmon, 4: yellow, 5: green, 6: blue, 7: purple, 8: pink). Solid lines indicate mean values and ribbons indicate the 95% confidence intervals. The dashed lines denote the critical epidemic threshold (Rt = 1). (TIFF) [file pntd.0011536.s004.tiff]

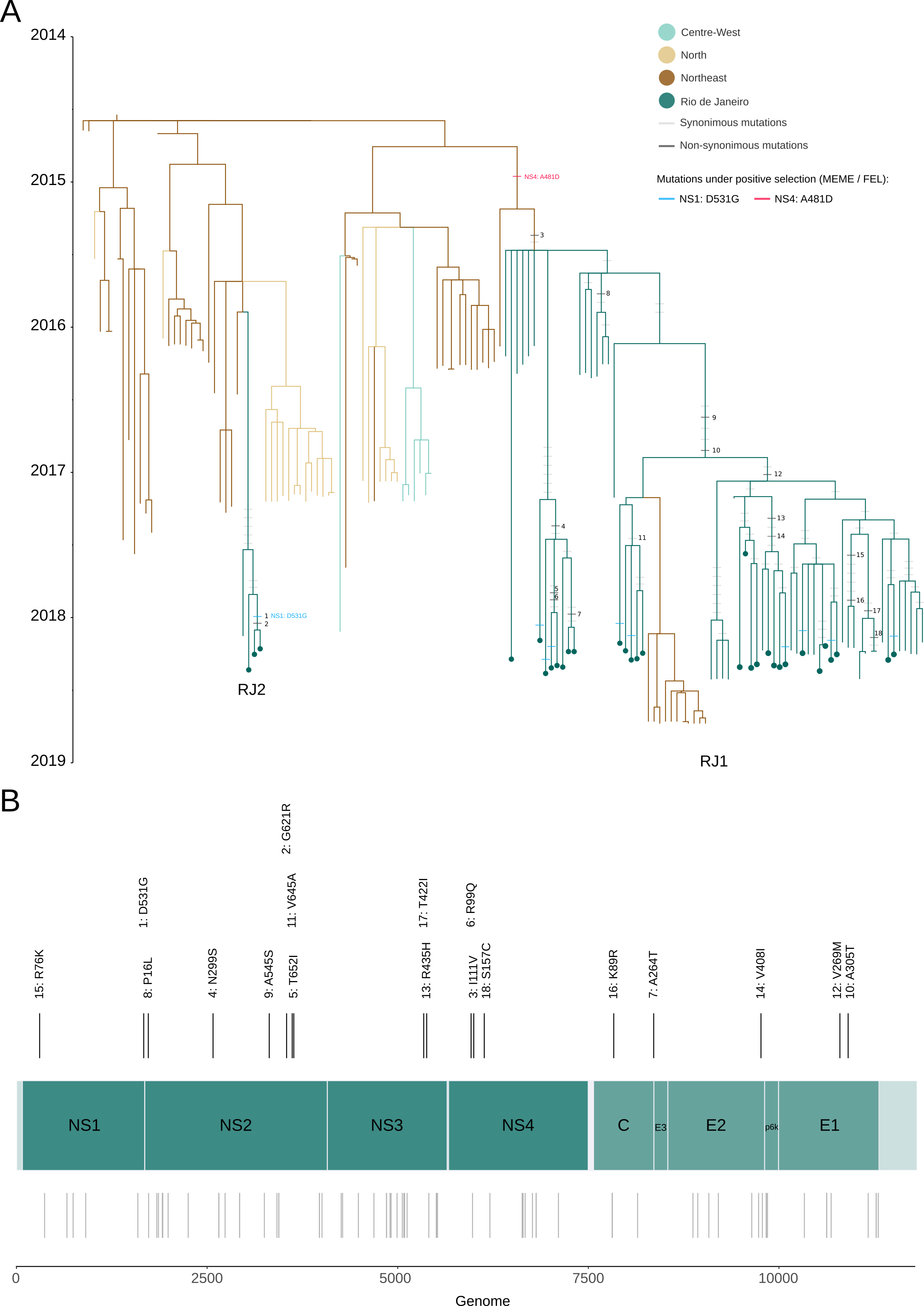

Supplement: S5 Fig — (A) The molecular clock tree had its branches colored according to the ancestral location’s reconstructions. Tip shapes indicate sequences generated in this study. Light gray ticks along branches indicate synonymous mutations, while dark gray ticks mark non-synonymous mutations. Mutations for which evidence of positive selection was found (MEME and FEL models) are marked in red (NS4: A481D) and blue (NS1: D531G). (B) CHIKV genome scheme exhibiting all mutations reconstructed and associated with RJ clades. Non-synonymous mutations are numbered from 1 to 18 and their positions and corresponding amino acid replacements are indicated by black vertical lines. Similarly, vertical gray lines indicate the position of the synonymous mutations inferred. Non-structural and structural open reading frames are colored in dark green and light green, respectively. (TIFF) [file pntd.0011536.s005.tiff]
