# Supplementary material for: Epidemiological and genomic investigation of chikungunya virus in Rio de Janeiro state, Brazil, between 2015 and 2018
Source: PLoS Negl Trop Dis. 2023 Sep 28;17(9):e0011536. doi: 10.1371/journal.pntd.0011536 (PMC10564160; doi:10.1371/journal.pntd.0011536)
Supplement: Attachment — Submitted filename: CHIKV_Rio_2014-2018_PNTD_review_20230624.docx [file pntd.0011536.s010.docx]

**Review**

Dear editors,

We thank you for your reply and the referee’s suggestions that we believe have improved our manuscript. We are encouraged by the review of the manuscript by Moreira et al.,“ Epidemiological and genomic investigation of chikungunya virus in Rio de Janeiro state, Brazil, between 2015 and 2018”. We complied with nearly all suggestions provided by the reviewers and commented on each of them in detail. Specifically, we emphasized the main novelties of our study, provided detailed descriptions of analyzed datasets and addressed the validity of selected priors for the phylogenetic analysis. As requested, we also created a GitHub repository with all data (input), code and output for each analysis in the manuscript (https://github.com/filiperomero2/CHIKV_RJ_2015-2018).

Below, we addressed each reviewer’s comment. The reviewer comments are in italics followed by our responses. All the changes are highlighted in red in the revised manuscript.

Thanks again for this opportunity, and we hope that the revised manuscript is now acceptable for publication in PNTD.

Best regards,

Dr Renato Santana Aguiar

**—**

*Reviewer #1: Moreira et al. investigated the transmission dynamics of the chikungunya virus in Rio de Janeiro state, Brazil, between 2015 and 2018 in this study.*

*Minor comments:*

*Could the authors provide an explanation for why they chose the strict molecular clock model and a flexible skygrid tree prior for the Bayesian phylogenetic analyses?*

R:

We thank the reviewer for the opportunity to provide clarification. We selected the skygrid prior due to its non-parametric nature. That is, this tree prior does not assume the virus population demography followed a pattern consistent with a specific function, as parametric priors do (e.g., coalescent constant/exponential growth). Therefore, given its flexibility and the timespan comprehended by the main dataset (four years/epidemic seasons), we expect the skygrid prior to result in more accurate (less biased) estimates.

In regards to the molecular clock model, we opted for the strict clock due to the fact the dataset comprehends a single viral lineage (CHIKV-ECSA Americas) sampled over the period of four years, with no clear signals of rate heterogeneity. This can be observed in the root-to-tip regression exhibited in Figure 3C, which displayed a high correlation between sampling dates and genetic divergence (correlation coefficient: 0.85, R-squared: 0.72). Also, stringent model selection analyses performed in previous studies that examined datasets of the same viral lineage (sampled over a similar timespan), indicated the strict molecular clock as the best fit model (Naveca et al, 2019; Xavier et al, 2019).

Finally, we repeated our main timescale analysis with the uncorrelated relaxed clock model (UCLN) and obtained results consistent with the strict molecular clock model. Both models infer the root of the tree in middle 2014 (strict 95% BCI: 29 May - 25 August; UCLN 95% BCI: 1 June - 25 August), while the clade RJ1 was dated to mid-2015 (strict 95% BCI: 4 April - 24 September; UCLN 95% BCI: 11 April - 5 October) and RJ2 to mid-2017 (strict 95% BCI: 12 March - 6 November; UCLN 95% BCI: 19 March - 22 November).

As the coefficient of variation of the UCLN model presented great uncertainty and included extremely low values (95% BCI: 6.68 x 10^-4^ - 0.43), we opted for keeping the analysis with the strict model as a key result, and to add the analysis with the UCLN model as supplementary result (Supplementary File S2). Modifications in the text were performed accordingly (page 14, lines 10-12; page 19, lines 11-14).

*Could the authors include more information about the various datasets they constructed for each analysis?*

R:

We assembled a total of three chikungunya virus (CHIKV) genome sequence datasets. The first dataset comprehended the global chikungunya virus diversity, including all sequences longer than 8,000 nucleotides publicly available on NCBI (as of 31 May 2022). Sequences without collection date or country of origin were removed. This dataset (n = 1,262) spanned all viral genotypes and was used to reconstruct the tree in Figure 3A. (as described on page 12, lines 2-6, page 13, lines 1-5).

The second dataset comprehended all sequences belonging to the ECSA Americas clade, as identified in the first analysis. As the key goal was to contextualize in detail the novel genome sequences from RJ collected in 2018, reference sequences collected after this year were removed from the dataset. Non-Brazilian ECSA-American sequences were also excluded, as a preliminary analysis indicated they were not related to the new sequences. Sequences were further filtered based on a root-to-tip regression, which identified sequences whose divergence degrees were inconsistent with the molecular clock (outliers). Noticeably, some of these sequences represented extremely long branches in the phylogenetic tree, likely reflecting genome assembly artifacts. This key dataset (n = 148) was used to infer the phylogenetic tree reported in Figure 3B and its temporal signal analysis is displayed in Figure 3C. It was also used for the dated phylogeographic reconstruction in Figure 4A. (described on page 13, lines 6-22).

The previous analysis led to the identification of a large clade composed mostly of Rio de Janeiro sequences (RJ1). We were then prompted to investigate its demographic history with the skygrid model. Therefore, we assembled a third dataset comprehending the 63 Rio de Janeiro sequences from this clade and performed a BEAST analysis with it. The results from the demographic reconstruction are displayed in Figure 4B. (described on page 14, lines 21-23; page 15, lines 1-5).

To further clarify our dataset assembly strategy, we performed minor and informative modifications in the aforementioned sections (page 13, lines 3, 6-12, lines 21-22; page 14, lines 22-23; page 15, line 1).

*Although the authors have provided input and R codes for the epi-analysis, I would like to see all input, metadata, and output files for each generated figure (F1-4 and Fig S1-4) publicly accessible in a repository such as GitHub before acceptance. I hope the authors recognize that this is essential for open-access science and reproducibility.*

R:

We completely agree with the recommendation. All input, metadata, code and output files are now publicly available on a GitHub repository (https://github.com/filiperomero2/CHIKV_RJ_2015-2018). This information was included in the revised manuscript (page 9, lines 11-12; page 15, lines 4-5).

---------------------

*Reviewer #2: Moreira FRR et al. revealed the epidemic and evolutionary history of Chikungunya virus in Rio de Janeiro state from 2016 to 2018. This is a valuable approach, and this manuscript uses a dataset that has a lot of potential for future work. I have several concerns and comments about the present analysis.*

*1. Previous studies have documented the epidemiological and evolution characteristics of Chikungunya virus in the city of Rio de Janeiro. Therefore, the author needs to highlight the innovative points worth publishing in this article.*

*(1)	Souza, T.M.L., Vieira, Y.R., Delatorre, E. et al. Emergence of the East-Central-South-African genotype of Chikungunya virus in Brazil and the city of Rio de Janeiro may have occurred years before surveillance detection. Sci Rep 9, 2760 (2019). https://nam11.safelinks.protection.outlook.com/?url=https%3A%2F%2Fdoi.org%2F10.1038%2Fs41598-019-39406-9&data=05%7C01%7Cwmdesouz%40utmb.edu%7C7359e4cb061b435a341308db5d7ee238%7C7bef256d85db4526a72d31aea2546852%7C0%7C0%7C638206571769886196%7CUnknown%7CTWFpbGZsb3d8eyJWIjoiMC4wLjAwMDAiLCJQIjoiV2luMzIiLCJBTiI6Ik1haWwiLCJXVCI6Mn0%3D%7C3000%7C%7C%7C&sdata=ZD6LJNZoKWD06QjL6wzb0%2BCyePP%2B%2FX8NnpNNaxV3V6k%3D&reserved=0*

*(2)	Xavier J, Giovanetti M, Fonseca V, Thézé J, Gräf T, Fabri A, Goes de Jesus J, Lima de Mendonça MC, Damasceno Dos Santos Rodrigues C, Mares-Guia MA, Cardoso Dos Santos C, Fraga de Oliveira Tosta S, Candido D, Ribeiro Nogueira RM, Luiz de Abreu A, Kleber Oliveira W, Campelo de Albuquerque CF, Chieppe A, de Oliveira T, Brasil P, Calvet G, Carvalho Sequeira P, Rodrigues Faria N, Bispo de Filippis AM, Alcantara LCJ. Circulation of chikungunya virus East/Central/South African lineage in Rio de Janeiro, Brazil. PLoS One. 2019 Jun 11;14(6):e0217871.*

R:

We thank the reviewer for the opportunity to clarify the distinguished features of our study. The studies mentioned were centered on the analysis of new Chikungunya virus (CHIKV) sequencing data, mostly focusing on molecular clock phylogeographic reconstructions. We acknowledge these contributions to the field and properly cited the papers in multiple instances throughout our manuscript. Our study distinguishes itself from previous publications due to the fact that we explored additional sources of data - epidemiological, clinical and climatic - to perform an integrative assessment of CHIKV epidemiology in Rio de Janeiro.

Therefore, our study presents a number of novel analyses (absent in previous publications), including: 1) the estimation of basic and time-varying reproductive numbers, which are essential parameters to contextualize pathogen transmissibility; 2) the inference of a vector climatic suitability index (index P), which reflects vector probability of occurrence given climatic variables; 3) Molecular adaptation models, which detects instances of positive natural selection in genome sequences. Through these approaches, it was possible to identify that oscillations in CHIKV incidence and transmissibility occurred in synchrony with index P, indicating these parameters can be associated with vector dynamics. Moreover, complementing our Bayesian time scale analysis (which independently confirmed previous studies), we performed molecular adaptation scans and detected two mutations that likely evolved under positive natural selection (nsP1: D531G, nsP4: A481D). These results are entirely novel and describe CHIKV epidemiological dynamics in Rio de Janeiro in detail. Due to this reason, we believe our paper is distinguished from previous research, and that it is worth publication.

*2. More information would be obtained if the dynamic pattern of the Chikungunya virus could be further explored based on the kinetic mechanism model, which integrated the epidemiological data, environmental data and molecular data in one model. The authors should discuss this topic in depth.*

*Henderson AD, Kama M, Aubry M, Hue S, Teissier A, Naivalu T, Bechu VD, Kailawadoko J, Rabukawaqa I, Sahukhan A, Hibberd ML, Nilles EJ, Funk S, Whitworth J, Watson CH, Lau CL, Edmunds WJ, Cao-Lormeau VM, Kucharski AJ. Interactions between timing and transmissibility explain diverse flavivirus dynamics in Fiji. Nat Commun. 2021 Mar 15;12(1):1671.*

R:

We thank the reviewer for the recommendation. While we recognize that the usage of the kinetic mechanism model would definitely be informative on Chikungunya virus (CHIKV) epidemiological dynamics in Rio de Janeiro, we do not present any serological data or analysis in our study. As this type of data is a key source of information for the model, its analysis seems unfeasible at this time.

Notwithstanding, although we do not merge information from different sources of data into a single model, we performed a set of analyses with genetic, epidemiological and climatic data that ultimately improved the understanding of CHIKV dynamics in the state. First, beyond supporting previously published phylogeographic reconstructions, our analyses reveal two CHIKV ECSA-Americas mutations that likely evolved under positive selection, nsP1: D531G and nsP4: A481D), which possibly have implications for virus epidemiology. These mutations are putative targets for functional studies *in vitro* and *in vivo*, which may reveal their phenotypic impact and epidemiological consequences. Second, our study is the first to report estimates of basic and time-varying reproductive numbers for CHIKV in RJ, contextualizing its transmissibility in comparison to other circulating arboviruses, such as Dengue and Zika viruses. Third, we showed that trends in transmissibility are highly consistent with mosquito climatic suitability, as measured with index P.

Altogether, our analyses highlight that CHIKV dynamics in Rio de Janeiro is determined by a complex interplay between virus molecular evolution, mobility, climate and vector ecology. We believe further studies analyzing serological, entomological and behavioral data would further benefit our understanding of CHIKV epidemiology in Brazil, (as stated on page 22, lines 20-23)	.

*3. The authors analyze the possible causes of low prevalence of Chikungunya virus in 2017 compared with 2016 and 2018, in the discussion section, and concluded that the trend observed in 2017 was unlikely to be caused by population immunity. Rather, it is more likely to be caused by environmental factors. Theoretically, the 2016 pandemic would have led to a reduction in the susceptible population, which in turn reduced the intensity of the pandemic in 2017. It is very important to better distinguish the role of each driver.*

R:

We agree with the reviewer's comment. Identifying and quantifying the relative contribution of different epidemic drivers is a current challenge in infectious diseases epidemiology. We argue that population immunity was an unlikely cause for the low incidence observed in 2017 because the virus ressurged in 2018 and the following years. Given that Chikungunya virus immunity is thought to be long lasting and that no major demographic event occurred, this indicates that a large pool of susceptible individuals was still available after 2016. Consistent with that idea, a seroprevalence study performed between July and October 2018 indicated a prevalence of 18% (95% CI: 14.8 - 21.2) for CHIKV (https://doi.org/10.1371/journal.pone.0243239). As the key distinguished feature of 2017 was related to variation in climatic variables, we hypothesized that these fluctuations could at least partially explain the trends in incidence, given their possible consequences for vector dynamics. To smooth our statement on the role of immunity, we added the term “primarily” to the sentence (“*Moreover, the large chikungunya epidemic in Rio de Janeiro in 2018 indicates that the trend observed in 2017 was unlikely to be caused primarily by population immunity.* ”, page 22, line 18).

*4. The author hypothesized that the low number of cases in 2017 can partially be explained by climatic factors since Ae. aegypti suitability, mean temperature, or humidity in 2017 was lower compared to previous and following years (page 22). The authors only presented the index P in Fig.1, however, there was no detailed description of the index P and its relation to Rt value in the Result part. Besides, the author mentioned the potential drivers may be mean temperature, or humidity. Can the author place the time-series of mean temperature, humidity and precipitation in the supplementary material?*

R:

We complied with the recommendation and inserted a new supplementary figure S3 displaying the time series of climatic variables (2m temperature, total precipitation and relative humidity) (page 17, line 6; page 33, lines 14-16). The numbers of other supplementary figures were adjusted accordingly.

*5. The line number is missing in the manuscript.*

R:

We thank the reviewer for notifying this issue. Line numbers were included in the revised version of the manuscript.

*6. The results regarding to the clinical presentation and the genome sequencing appear discordant when placed together.*

R:

We apologize, but we could not identify the points of discordance between clinical and sequencing data.

---------------------

*Reviewer #3: The authors did a great job in analyzing the impact of CHIKV during an outbreak in Rio de Janeiro using scalable data such as R(o) and R(t). The authors have brought up under introduction that urbanization is a contributor to CHIKV transmission and its diversity. Adding additional statements (or analysis) under discussion such as spatial analysis stratified by regions within Rio would enrich the conclusions, providing insight into the socioeconomic determinants that may contribute to the overall transmissibility of CHIKV-ECSA in a hyper-urbanized city such as Rio de Janeiro.*

R:

We thank the reviewer for commending our work. Unfortunately, epidemiological data stratified by regions in the city of Rio de Janeiro is not available at this time, making the analysis unfeasible. Notwithstanding, while we recognize this line of inquiry would be a great addition to the research on CHIKV epidemiology in Brazil, we believe it is beyond the scope of our study.

*Introduction - "Chikungunya's geographic distribution – particularly of Asian, IOL and ECSA lineages has been expanding rapidly over the last 20 years probably due rapid urbanization, globalization of trade, and virus evolution and adaptation to local variation in the distribution of vector species"*

*• To check for a more fitting reference for this statement*

*• "Due to air and fluvial connectivity and high mosquito climatic suitability" - To clarify on the suitable climatic conditions in Rio de Janeiro*

R:

We thank the reviewer for the recommendations. We added additional references to the sentence mentioned and included information on climatic suitability for arboviruses in Rio de Janeiro (page 5, line 21; page 6, line 16; page 26, lines 19-25).
